# Reply to: "Concerns about cognitive performance at chance level"

**DOI:** 10.1038/s41598-021-93954-7

**Published:** 2021-07-30

**Authors:** Adam J. Toth, Mark J. Campbell

**Affiliations:** 1 Lero, The Irish Software Research Centre, University of Limerick, Castletroy, Limerick, Ireland; 2 Department of Physical Education and Sport Sciences, University of Limerick, Castletroy, Limerick, Ireland

**Keywords:** Human behaviour, Cognitive control

**replying to**: L. Jost; *Scientific Reports* 10.1038/s41598-021-93953-8 (2021).

## Introduction

The following is a response to Jost^1^, who contests findings from a recently published mental rotation study^2^. In the original study, participants scored 2.765 ± 0.182 out of 20, when scored such that a point was awarded when both ‘same’ images on the V&K MRT^3^ (Vandenberg and Kuse Mental Rotations Test) were correctly identified in a given trial. These scores indeed are close to chance, with Hegarty^4^ defining chance performance to be 3.33 out of 20 when using the same scoring method. In his critique, Jost^1^ poses two questions regarding the findings presented by Toth and Campbell^2^. Firstly, he asks how it is possible that participants performed this poorly and secondly, whether one can meaningfully analyze performance at chance level.

In his hypothesis to his first question, Jost^1^ cites that performance at chance level may suggest a possible misunderstanding of the task by participants, causing them to perform a different cognitive task than that of mental rotation. However, in the original study by Toth and Campbell^2^, it is outlined that participants were presented clear instructions on how to perform the task and were also presented with three practice sample trials. This is in accordance with best practice and previous work using the V&K MRT^3^ to assess mental rotation ability (see^5,6^ for examples). This line of reasoning around misunderstanding we argue is thus highly unlikely.

Jost^1^ also poses that, while possible that participants were simply poor at mental rotation tasks, this is highly improbable as according to Jost^1^ “in no mental rotation study have adult participants ever performed even close to this poorly”. However, several previous studies have demonstrated poor to very poor performance on the V&K MRT in multiple samples^5–8^ with Kruger and colleagues even reporting low performance among security screeners despite the removal of ‘more difficult questions’. We argue it is also very plausible that studies in which participants scored very poorly on such a test previously may have ended up in a file drawer rather that published. Despite the poor performance of participants in this study, it is important to publish unconventional findings to mitigate publication bias when experimental methodology is deemed sound, which was the case here. Moreover, Jost^1^ agrees himself that the converging evidence of gaze behaviours, pupillometric and reaction time data presented by Toth and Campbell^2^ suggest that it is plausible that cognitive resources were over taxed, evidencing the difficulty participants had with the test which may have led them to guess at the end of trials, resulting in the overall observed poor performance. However, determining with certainty the presence of guessing and potential for malingering can be very difficult, with previous research showing that the performance of those specifically instructed to guess in forced choice tests can overlap those who display genuine low performance^9^. The low performance displayed by both sexes in this study may also explain the lack of sex differences present, where both^6,10^ cite that sex differences may be most distinguishable when examining individuals who are intermediate at mental rotation, as opposed to those who are inexperienced or are trained experts.

Furthermore, our lab is targeting a large study this year examining differences between the Shepherd and Metzler and Vandenberg and Kuse mental rotations tests and the quality of the stimuli used, and we hope to conclude on best practice regarding administration/scoring/recording of moderator variables. For the purposes of this discussion, we have provided recent evidence of poor performance on the V&K MRT and highlighted its potential drawbacks as a tool to purely investigate spatial cognition (additional recent work showing also poor performance is conducted by^8^, however it must be noted that it is not below chance). We also highlight that despite our overall scores being below chance level, it cannot be concluded as to whether all/some/any of the trials were actually guessed upon at this point. We have highlighted this very point in our response in addition to the fact that previous low performance, which may have been in opposition to the existing literature, may have been subject to the file-drawer phenomenon and thus, publication bias. We think it a testament to *Scientific Reports* for allowing this debate and acknowledging the transparency of our sound methodology, technical quality and data sharing, and while we have nothing further to add but conjecture to the above point, we certainly acknowledge that further work should be done in an attempt to replicate and/or confirm the findings presented in the original work.

Also, Jost^1^ argues that the evidence of increased cognitive effort displayed in our study does not suggest that the effort was invested toward mental rotation, we counter this point by stating that it is likely that mental rotation skills were taxed due to the test laid in front of participants requiring this specific cognitive ability. Our use of maximum pupil diameter in this regard is also the most robust eye-tracking metric of cognitive effort as we have laid out in previous work, as it does not fall victim to the fact that viewing times across stimuli were not controlled^11,12^. Indeed, the V&K MRT is arguably the most widely used and cited test of mental rotation since its introduction in 1978. This being said, we note that given that the V&K MRT^3^ requires between 3 and 5 mental rotations to be held in memory when completing a given trial, the test is unique to other mental rotation tests in that it may engage additional cognitive abilities in addition to pure mental rotation ability^2^. Thus, it may be that taxed memory and not spatial or mental rotation ability is explaining the poor test performance among the participants who took part in the study. The potential for engagement of numerous cognitive skills speaks to the quality of the V&K MRT as a pure measure of mental rotation and general spatial ability. Previously, when testing a similar cohort using the original MRT designed by Shepherd and Metzler^13^, Toth and Campbell^2^ found pupillometry to differentiate performance between sexes with overall behavioural performance corroborating previously reported performances^11^.

It is also worth noting that many factors can influence mental rotation performance, including sleep, motivation, etc. In the study by Toth and Campbell^2^, many of these factors were not controlled for and future research may further illuminate the mechanisms underlying mental rotation performance, specifically on the V&K version of the MRT. Overall, there are numerous plausible explanations for why participants may have performed poorly on the V&K MRT despite their best effort to engage in the cognitive process of mental rotation. In line with the ethos of Scientific Reports we report a study which we consider to be high in technical quality, complete with robust methodological, analytical and statistical analyses. Furthermore, we were transparent and honest in explaining potential limitations of our study and poor performance by participants in our discussion section. To this end, we provide our data in the OSF repository for those who may wish to further investigate the issues discussed here^14^.

The additional psychophysiological measures utilised by Toth and Campbell^2^ in their work investigating sex differences on the V&K MRT provide unique and additional insight into the mental process employed by participants in their attempt at completing the MRT, the poor observed performance by participants on the task, and the quality of the task itself. In the original study, the addition of eye tracking and pupillometric data by Toth and Campbell^2^ indicates the high degree of cognitive effort employed by participants and speaks to the difficulty they had in completing the V&K MRT. Future work may not only compare the efficacy of the V&K and S&M MRTs for evaluating mental rotation performance, but may also specifically establish the neurophysiological correlates of random guessing behaviour on tests of mental rotation, so that the process may be better identified and controlled for in future spatial cognition research.

So how does below chance performance impact the central claims of our paper?

The following are the central claims of our original paper, which evaluated performance on a computerized version of the V&K MRT, and investigated, using novel pupillometry and gaze measures, the purported sex difference in performance on this task.

1. Firstly, we found no significant performance difference between males and females on the V&K MRT. As such, our original claim was that our “study provides further evidence that the nature of sex differences on the V&K MRT is complex, with a number of factors potentially involved beyond mental rotation ability.” Whilst the below chance performance poses a limitation, in our view this original observation is still valid, and finds support in some of the previous literature^15–18^. In addition, the difficulty in determining the incidence of guessing—given the nature of combining multiple response per trial—further highlights the drawback of this test compared to the S&M MRT, where malingering can be much more easily established.
2. Second, we showed that all participants displayed large increases in pupil diameters during completion of the MRT, evidence of the cognitive demand of the task. This highlights that “unlike the S&M MRT, pupillometry measures indicate that the V&K MRT may be exceedingly difficult for participants of both sexes who do not have an educational background associated with superior spatial ability”. Regardless of whether the low performance observed here was due to guessing on none/some/all of the presented trials, both the behavioural and pupillometric data suggest that participants obviously found the test exceedingly difficult and again, calls into question the ability of the V&K MRT to truly evaluate spatial cognition in isolation of other mental processes.
3. Third, we discovered an association between fixation patterns and performance among all participants. In doing so, “we showed for the first time, that participants may be adopting a leaping strategy including the strategy as evidenced by their differential fixation patterns on mirror and structural foil trials.” This very clear pattern of fixation strategy was recorded and analysed separate to score and does suggest engagement in the instructed process of mental rotation, as opposed to random eye movements which would likely show no clear difference in focus of attention between trial and stimuli types. That being said, upon searching through PubMed and Google Scholar databases, the evaluation of gaze strategy differences during malingering and knowledgeable engagement on tests of cognitive ability is non-existent and could be a very attractive topic of research in the future.

We contend therefore that despite the below chance scores noted for our groups the central claims of our paper remain sound and valid. Additionally, we have discussed for a duration of 4 paragraphs the limitations of our paper acknowledging the poor scoring, our sample and differences to other samples and studies.

We thank Jost^1^ for raising his concerns and acknowledge their merit as it pertains to progressing this important field of study. Perhaps performance at or below chance level is guessing or malingering but we also see that it may be evidence of poor spatial cognition of our sample of Irish university students. We argue that this test performance is valid and worthy of examination as do others^19^ and that although unconventional, these behavioural and physiological data are noteworthy, and their publication mitigates the bias present among research in many scientific fields. In addition to the provision of our data from the original study to the OSF repository^14^, where further interest may be directed, we endeavour next to pre-register a study or series of studies which follows to repeat our methods with another Irish sample and are seeking interested parties and labs to join us in collecting other cohorts from different populations/ countries to robustly seek clarification on this test of mental rotation.
